# Changes in stool frequency following chicory inulin consumption, and effects on stool consistency, quality of life and composition of gut microbiota

**DOI:** 10.1016/j.foodhyd.2019.06.006

**Published:** 2019-11

**Authors:** Anthony W. Watson, David Houghton, Peter J. Avery, Christopher Stewart, Elaine E. Vaughan, P. Diederick Meyer, Minse J.J. de Bos Kuil, Peter J.M. Weijs, Kirsten Brandt

**Affiliations:** a NU-Food Research Facility, Human Nutrition Research Centre, Newcastle University, NE1 7RU, Newcastle upon Tyne, UK; b Wellcome Trust Centre for Mitochondrial Research, Institute for Cell and Molecular Biosciences and Institute of Neuroscience, Newcastle University, NE2 4HH, Newcastle upon Tyne, UK; c School of Mathematics & Statistics, Newcastle University, NE1 7RU, Newcastle upon Tyne, UK; d Institute of Cellular Medicine, Newcastle University, NE2 4HH, Newcastle upon Tyne, UK; e Sensus (Royal Cosun), Borchwerf 3, 4704 RG Roosendaal, the Netherlands; f Department of Nutrition and Dietetics, Amsterdam University of Applied Sciences, Postbus 2165, 1000 CD Amsterdam, the Netherlands

**Keywords:** Inulin, Digestive health, Microbiology

## Abstract

Inulin is a soluble dietary fibre, also classified as a prebiotic, extracted from chicory roots. The present study aimed to determine the effect of consumption of native chicory inulin on the stool frequency of middle-aged to older adults (40–75 years old) with uncomfortably but not clinically relevant low stool frequency, specified as two to four days without bowel movements per week. Two randomised, double blind, placebo-controlled crossover trials were conducted using similar protocols in differing populations. Trial A was conducted in Amsterdam, The Netherlands and subsequently Trial B was conducted in Newcastle, United Kingdom. Both trials involved supplementation for 5 weeks with 10 g per day of inulin or placebo, a washout period of 2 weeks, and then crossed over to receive the other treatment. In Trial B, faecal gut microbiota composition was assessed using 16S rRNA gene sequencing. In Trial A, which 10 volunteers completed, the stool frequency was significantly increased to an average 4.9 ± 0.23 (SEM) times per week during inulin periods versus 3.6 ± 0.25 in the periods with placebo (*p* = 0.01). In contrast, in Trial B which 20 volunteers completed, there was no significant effect of the inulin on stool frequency (7.5 ± 2.1 times per week with inulin, 8.1 ± 3.0 with placebo, *p* = 0.35). However, many subjects in Trial B had a stool frequency >5 per week also for the placebo period, in breach of the inclusion criteria. Combining the data of 16 low stool frequency subjects from Trials A and B showed a significant effect of inulin to increase stool frequency from 4.1 to 5.0 per week (*p* = 0.032). Regarding secondary outcomes, stool consistency was significantly softer with inulin treatment compared to placebo periods, it increased 0.29 on the Bristol stool scale (*p* = 0.008) when data from all subjects of Trials A and B were combined. No other differences in bowel habit parameters due to inulin consumption were significant. None of the differences in specific bacterial abundance, alpha or beta diversity were significant, however the trends were in directions consistent with published studies on other types of inulin. We conclude that 10 g per day of native chicory inulin can increase stool frequency in subjects with low stool frequency.

## Highlights

• Inulin as 10g day^-1^ for 5 weeks significantly increased stool frequency in 16 older volunteers compared with maltodextrin..
• This increase was found in volunteers with low stool frequency (2 days or more per week without bowel movements).
• Directions of changes in secondary outcomes were generally consistent with expectations for prebiotic effects of inulin.
• Secondary outcomes included quality of life symptoms, stool consistency and composition of the faecal microbiota.
• Among secondary outcomes, only softening of stool consistency reached significance within the present study.

## Graphical abstract

**Figure:**
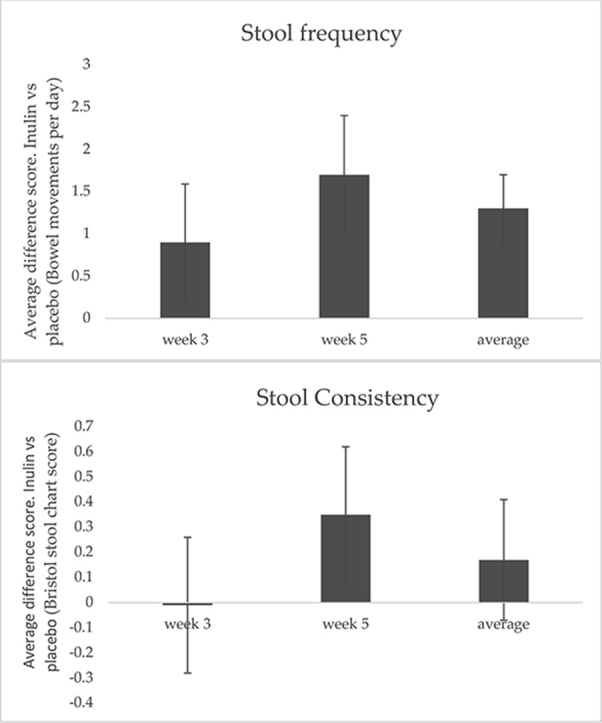

## 1 Introduction

Constipation is a gastrointestinal tract (GIT) symptom that is characterised by irregular, difficult and/or painful stool expulsion and hard, dry stool consistency (Mccrea et al., 2009), and can cause additional GIT symptoms such as distention, abdominal pain and poor appetite, amongst others (Lembo & Camilleri, 2003). The prevalence of constipation is difficult to determine as most people do not seek medical treatment, however, it is estimated to cause recognised discomfort to around 2–27% of the general population, with an increase with age (Gallagher & O'mahony, 2009).

Although the exact aetiology of constipation remains unknown, Suares et al. (2011), amongst others, has identified that low levels of physical activity, medication, depression and dietary composition are all likely to contribute to the incidence of constipation. Specifically, consuming less than the recommended daily fibre intake of 25 g/day has been associated with an increased incidence of constipation (Suares & Ford, 2011). In a recent meta-analysis, increased dietary fibre has been shown to be a useful treatment in those people suffering with constipation (Rao, Yu, & Fedewa, 2015), although how to increase dietary fibre intake remains an area of concern, and it is not known if all types of fibres have equal effects.

Inulin is a soluble dietary fibre that occurs as a storage polysaccharide in a variety of plants (Schaafsma & Slavin, 2015). A specific subset of dietary fibres, such as inulin, can selectively stimulate certain gut microbiota species and those fibres may be termed prebiotics. The most recent prebiotic definition of the International Scientific Association of Pro- and Prebiotics (ISAPP) is *“a substrate that is selectively utilised by the host microorganisms conferring a health benefit”* (Gibson et al., 2017). Inulin, derived from the chicory root, is well established for its prebiotic properties, as recognised by ISAPP. Inulin fibre passes through the upper GIT undigested, reaching the colon where it is fermented by bacteria thereby modulating the bacteria residing in the GIT, i.e. ‘gut microbiota’ (Gibson & Roberfroid, 1995). Inulin has been shown to improve bowel habit through increased stool weight, softer stools, reduced GIT symptoms, and improved quality of life (Castiglia-Delavaud et al., 1998; Den Hond, Geypens, & Ghoos, 2000; Vandeputte et al., 2017a). Although the exact mechanism of how inulin may improve GIT health and relieve constipation are not completely understood, the impact of nutrition (Flint, Scott, Louis, & Duncan, 2012), and more recently inulin's ability to modulate the gut microbiota have been suggested to play a role (Vandeputte et al., 2017a).

The gut microbiota has been shown to contribute towards nutrition, metabolism, immune response, intestinal architecture, and GIT health (Hooper & Gordon, 2001). Changes in ‘normal’ bowel habits, commonly reported in people with constipation, have been linked to changes in gut microbiota composition, which has been suggested to have a functional role in the pathogenesis of constipation (Kim et al., 2015; Zhu et al., 2014). Consumption of foods high in fibre has been shown to be an effective treatment for symptoms of constipation (Lembo & Camilleri, 2003; Voderholzer et al., 1997). Fibre provides a substrate for microbial fermentation, and in so doing stimulates the growth of intestinal microbiota (Flint et al., 2012). Modulating the gut microbiota through fibre intake has the potential to increase the abundance of potentially beneficial bacteria, whilst reducing potentially pathogenic bacteria (Chen et al., 2013), and this may alleviate the symptoms of constipation (Ingvar, 2004). Intake of native chicory inulin has previously been shown to stimulate changes in the relative abundance of *bifidobacteria* in several studies using culturing or quantitative PCR (Schaafsma & Slavin, 2015), and secondly mixes of chicory inulin type fructans show similar effects using 16S rRNA sequencing approaches (Reimer et al., 2017). More recently inulin has been shown to increase *Bifidobacterium*, *Bilophila* and *Anaerostipes* in constipated subjects (Vandeputte et al., 2017a).

The primary aim of the current study was to investigate the effect of native chicory inulin on stool frequency in healthy middle-aged and older adults, albeit with self-reported unsatisfactory bowel movements. Secondary aims were to assess associated effects on other aspects of GIT function, specifically stool consistency; quality of life; and gut microbiota.

## 2 Materials and methods

### 2.1 Subjects

Subjects were recruited for Trial A, in Amsterdam, Netherlands, or Trial B, in Newcastle upon Tyne, United Kingdom, with circulation of flyers, visits to general practitioners, and advertisements in regional and local newspapers. Volunteers received a comprehensive information brochure and were assessed for eligibility based on the exclusion and inclusion criteria by completing a questionnaire, which was confirmed at a screening visit by an independent physician, nurse or research technician. At the screening visit, subjects were provided a complete oral explanation of the purpose and procedures of the trial, and questions were addressed. After the questionnaire was reviewed to decide on the inclusion of the subjects, signed written informed consent was obtained from all subjects before any protocol-specific procedures were carried out. The studies were reviewed and advised by the Ethics Committees of the Independent Review Board of Amsterdam and the Faculty of Science, Agriculture and Engineering of Newcastle University, respectively. Trials A and B were registered under CCMO as NL27269.003.09 in Amsterdam and as ISRCTN97558933 in Newcastle, respectively.

***Trial A****.* The number of subjects recruited for trial A was based on stool frequency data from a similar cross-over trial (Den Hond et al., 2000) (Table 1), showing that 22 subjects would provide a power of 90% to detect a difference between groups of 1.5 in weekly stool frequency with alpha 5%. Sixteen volunteers were recruited for the study and 10 subjects, who met the criteria and submitted all requested records, completed the study. Both the average and median age was 52 years, ranging from 45 to 62 years.

***Trial B****.* Trial B was powered based on the stool frequency data from trial A, showing that 17 volunteers completing the study would be necessary to determine a difference of 1.3 at the 5% significance level with a power of 90%. With an assumed drop out percentage of 30%, the anticipated enrolment was 22 volunteers. However, recruitment continued for slightly longer, in order to increase the number of male volunteers. Twenty-six volunteers were recruited, 21 female and 5 male, 20 of which completed the study (16 female and 4 male). Both the average and median age was 62 years, ranging from 51 to 74 years.

***Inclusion criteria:*** Inclusion criteria for both trials were both healthy men and women aged between 40 and 75 years inclusive with self-reported low stool frequency, i.e. 2 days or more per week without successful bowel movements.

***Exclusion criteria:*** Volunteers were excluded from the study if they had a clinical diagnosis of constipation as defined by the Rome criteria (Longstreth et al., 2006); had anatomical causes of reduced bowel function; a history of colonic or anal surgery; or inflammatory bowel diseases (Crohn's disease, ulcerative colitis). Subjects that had major surgery or current or recent (<5 years) diagnosis of cancer, coronary heart disease or diabetes were also excluded. Other exclusion criteria included current or recent medication including the use of opium preparations, antibiotics, laxatives or diuretics in the 3 months prior to study commencement that may influence constipation symptoms and/or gut microbiota composition, or any other condition that in the view of the volunteer's general practitioner or the study manager may make the volunteer unsuitable for the trial (see further details in Supplementary Document 1).

### 2.2 Study design

**Fig. 1:**
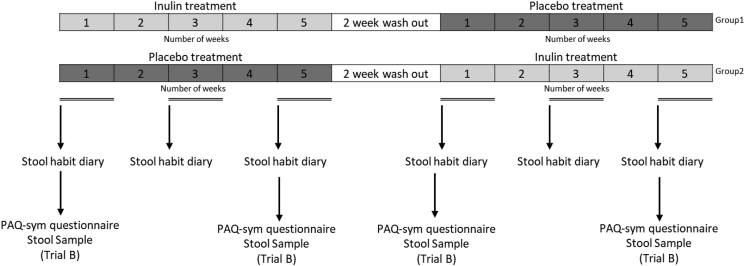
Trial design for A and B. Numbers 1–5 denote the number of weeks during the treatment phase. **(**PAQ-sym - Patient Assessment of Constipation Symptoms). Measures which were only conducted in trial B be are labelled “trial B” (PAQ-sym questionnaire and stool sample).

Both studies used a randomised and double-blind, cross-over placebo-controlled trial design. In the first treatment period, half of the subjects received either inulin or placebo respectively and this was reversed in the second treatment period (Fig. 1). The duration of each treatment period was 5 weeks and a wash-out period of at least 2 weeks followed the first treatment period (Fig. 1).

### 2.3 Study products, dosing schedule and diet during the trial

The trial used Frutafit^®^ inulin HD as produced by Sensus BV (Roosendaal, the Netherlands) from chicory roots. Frutafit^®^ inulin has been confirmed as a safe ingredient through its formal review of Sensus’ GRAS documentation (FDA, 2003). The placebo maltodextrin (MD 20) was obtained from Avebe (Foxhol, the Netherlands). Maltodextrin is a fully digestible carbohydrate, not a fibre. Maltodextrin behaves similarly to inulin in solution and tasting behaviour thus the placebo drink was identical in taste and texture to the inulin drink. Each volunteer received all the treatment materials in two batches, one batch at the start of each treatment period. In Trial A, each subject received 80 pre-mixed orange juice drinks containing 5 g of either inulin or placebo (enough for up to 40 days); the subjects were suggested to consume one drink in the morning and one drink mid-afternoon. In Trial B, each subject received 80 sachets with each 5 g inulin or placebo and a package with 80,100 ml bottles of shelf-stable juice or water (according to preference), and a mixing container. The orange juice used for this trial was selected as it has previously been shown not to have any pre-biotic effects (Hickey, Calloway, & Murphy, 1972). The subjects were instructed to mix one sachet and one portion of the liquid in the mixing container, and drink the mixture within 2 h of mixing, twice a day with breakfast and mid-afternoon. Volunteers used 1 portion of juice mix per day in the first three days when starting the study. After 3 days and during the rest of the trial the volunteers were instructed to consume 2 portions per day to reach the 10 g dose, and to return any unused study product to the study site (for compliance monitoring).

Throughout the trial, volunteers were asked to follow their normal diet and exercise habits and to try to avoid changes during the study, other than to avoid foods containing prebiotics and probiotics, which included foods containing inulin-type fructans (Examples Supplementary Document 1). Compliance to the recommended diet was confirmed verbally at each post randomisation study visit.

### 2.4 Randomisation and blinding

**Fig. 2:**
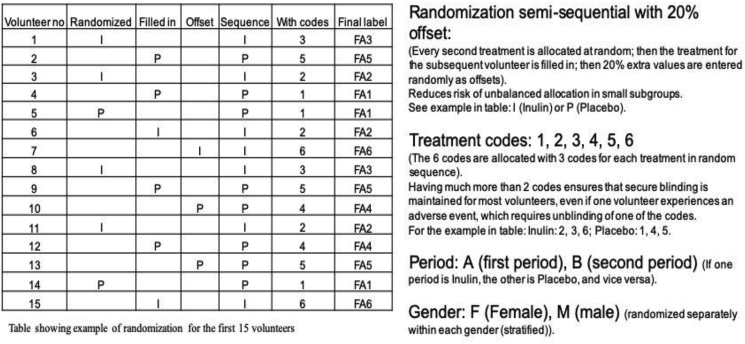
Illustration of principle of semi-sequential randomisation with offset. (Text in brackets explains how it was done), other text explains the purpose of the corresponding step.

At the start of the first 5-week treatment period, volunteers were randomly assigned to either inulin treatment or placebo. In Trial A, volunteers were randomised into the two groups by the study team. In Trial B, randomisation was stratified by gender using a computer-generated semi-sequential multiple-coded approach (Fig. 2), where each of the 2 treatments were allocated 3 different codes, and the allocation balanced to near equal proportions for every 6 volunteers.

This provided two specific benefits, compared with a simple random allocation of each volunteer into one of the two groups: a) If an adverse event required unblinding of one volunteer, this would not automatically unblind all the other volunteers as well; b) Even with relatively low volunteer numbers in one of the genders, the probability of substantially different numbers of subjects allocated to the two treatments within a gender was much lower than if simple random allocation was used (detailed description of the algorithm is in Supplementary Document 1, the macro is included as an Excel file, Supplementary Document 2). Study sachets were prepared by Sensus BV in pre-packed boxes pre-labelled with the subject numbers and study phase (see ‘Final label’ in Fig. 2). Subjects and all research staff involved in the conduct of the study were thus verifiably blinded to the treatment allocation, i.e. the sequence of study product intake, until final database lock. For safety reasons, sealed envelopes specifying the treatment corresponding to each of the different treatment labels were kept with the research team, to allow emergency unblinding if necessary. After the trial, Sensus BV was able to verify that all seals were intact, except for the agreed unblinding of the code corresponding to the non-urgent adverse event mentioned below.

### 2.5 Subject data collection and questionnaires

During weeks 1, 3 and 5 of each study period, volunteers were required to fill in bowel habit diaries for both Trials A and B. The bowel habit daily diary for Trial A included self-assessment of seven parameters, whereby each day the subject noted the time of each defecation event, stool consistency (Bristol stool scale), as well as scores from 1 to 4 (normal, reasonable, high, very high) for intestinal symptoms (ease of defecation, flatulence, rumbling, bloating and cramps) as previously described (Alles et al., 2007). In Trial B, the bowel habit daily diary was modified by removing a question on ‘ease of defecation’. Additionally, a validated questionnaire Patient Assessment of Constipation - Symptom (PAC-SYM) (under licence from Mapi Research Trust, France) was provided to the volunteers to be completed prior to and following the study period. Although this questionnaire has been developed for patients with a clinical diagnosis of constipation, it includes a wide range of symptoms with varying severity, and therefore is relevant for the healthy volunteers recruited to the present study. The corresponding Quality of Life questionnaire (PAC-QoL) was not used, since the ranges used for the outcomes were strongly skewed towards hospitalised patients, which was not relevant for this study population. Adverse events and concomitant medication were recorded for the duration of the trials in accordance with ICH/GCP guidelines. One volunteer experienced a migraine episode during an inulin consumption period. However, in consultation with the volunteer's GP it was concluded that the event was not related to the treatment.

### 2.6 Bacterial DNA extraction and 16S rRNA gene sequencing bacterial profiling

Volunteers from Trial B were asked to provide a stool sample prior to commencing the inulin or placebo treatment and again after 5 weeks supplementation, and after the washout period immediately prior to starting the second arm of the study; this was repeated upon completing the cross-over (Fig. 1) (four stool samples in total). Each stool sample was homogenised at room temperature using a mortar and pestle as previously described (Vandeputte, Tito, Vanleeuwen, Falony, & Raes, 2017b), and a 5 g aliquot was stored at −80 °C until DNA was extracted in order to minimise alterations in bacterial composition following 16S rRNA gene sequencing (Lauber, Zhou, Gordon, Knight, & Fierer, 2010). The samples were managed according to the requirements of the Human Tissue Authority (HTA). An aliquot of 300 mg of stool from each time point was extracted using MP FastDNA™ Spin Kit for Feces following the manufacturer's instructions (Qbiogene, MP Biomedicals, Illkirch, France). The V4 region of the 16S rRNA gene was amplified using barcoded Illumina adaptors primer pair 515F/806R and then sequenced in the MiSeq platform (Illumina) using the 2 × 250 bp paired-end protocol yielding pair-end reads that overlap almost completely. The primers used for amplification contain adapters for MiSeq sequencing and single-end barcodes allowing pooling and direct sequencing of PCR products (Caporaso et al., 2012).

16S rRNA gene pipeline incorporated alignment-based and phylogenetic approaches to maximise resolution of data. Paired reads were demultiplexed based on the unique molecular barcodes and then merged using the USEARCH v7.0.1090 (Edgar, 2010), allowing no mismatches with a minimum overlap of 50 bases. The merged reads were then trimmed at the first base with Q5, followed by a quality filter was applied and reads containing >0.05 expected errors were removed. The 16S rRNA gene sequences were then clustered into Operational Taxonomic Units (OTU), a term used to classify groups of bacteria that are closely related at a cut-off similarity value of 97% using the UPARSE algorithm (Edgar, 2013). The OTUs were mapped onto an optimised version of the SILVA database that incorporated only the 16S V4 region to determine taxonomies (Quast et al., 2013). Bacterial abundances were recovered by mapping the demultiplexed reads to the UPARSE OTUs. A custom script was then constructed for a rarefied OTU table using output files from the previous two steps, allowing analysis of phylogenetic trends, alpha-diversity and beta-diversity (Lozupone & Knight, 2005). Each sample was rarefied to 2997 reads, (range of 134–141,466). Four samples below this were removed for gut microbiota analysis. Raw data is publicly available through the short read archive database.

### 2.7 Statistical analysis

Data recorded in the bowel habit diaries during weeks 3 and 5 of each treatment period were transformed into difference data (by subtracting the average of the recorded week 3 and 5 scores during the placebo period from the average week 3 and 5 scores for the corresponding inulin period) in order to express the effect of the treatment as two numerical values for each volunteer. The overall trial average difference for each volunteer was also calculated, using the combined average of weeks 3 and 5 for the difference in outcome between the two treatments. These difference data were tested for (normal) distribution before being analysed for the statistical difference from zero using a one sample T-test. Additionally, for data that were recorded in a comparable way in both Trial A and Trial B (i.e. stool frequency, consistency, flatulence, bloating, cramping, rumbling), the overall trial average difference for each volunteer were analysed together, combining all volunteers in both trials. The values were analysed using ANOVA (General Linear Model in Minitab). This analysis distinguished between volunteers who during week 1 of their placebo period met the criterion of ≥ two days without bowel movements per week i.e. ‘low initial stool frequency’, and those who did not meet this criterion, thereby had less than two days without bowel movements i.e. ‘high initial stool frequency’. Due to the run-in dosing schedule in week 1 and anticipated increasing effect with inulin treatment over time, the bowel habit data recorded during week 1 were not used otherwise. The ‘low initial stool frequency’ criterion was met by 10 volunteers from Trial A and 6 from Trial B. The model factors were initial frequency (either low or high), and site (either Trial A or Trial B), nested within initial frequency, to determine the mean and standard deviation and significance corresponding to the hypotheses: (1) that the overall effect differs from 0; (2) whether the outcome differed among volunteers with low or high initial frequency (interaction with initial frequency); and (3) whether the outcome was affected by site among volunteers with low initial frequency. Where the outcome showed a significant interaction with the initial frequency, an additional one-sample T-test was used to analyse the data from volunteers with low initial frequency only. A Bonferroni correction was applied to the *p*-values for the 6 secondary outcomes (other than the primary outcome of change in stool frequency) resulting in α = 0.008, rather than the 0.05 for the primary outcome, The analysis and visualisation of bacterial communities was conducted using R (R-Core-Team., 2014). The phyloseq package was used to import data and calculate alpha and beta diversity metrics (Mcmurdie & Holmes, 2013). The Kruskal-Wallis test was used when comparing three or more categories. A Wilcoxon signed rank test was used to assess between-group differences for changes in relative abundance of bacteria at pre and post intervention. Correlations between two continuous variables was analysed using linear regression models, using p-values to indicate the probability that the slope of the regression is zero. Differences in beta diversity (weighted and unweighted Unifrac and Bray Curtis were assessed using PERMANOVA. All *p*-values presented were adjusted for multiple comparisons using the FDR algorithm (Benjamini & Hochberg, 1995). The effect size and significance of each covariate were determined using the ‘envfit’ function in ‘vegan’ comparing the difference in the centroids of volunteer, gender, inulin and time-point relative to the total variation (Oksanen et al., 2015). All *p*-values derived from envfit were adjusted for multiple comparisons using FDR adjustment. Ordination was performed using NMDS based on Bray–Curtis dissimilarity. The significance value was determined based on 10,000 permutations. Data obtained from the PAC-SYM questionnaire (Trial B only) were added up into domain scores (abdominal, stool, rectal, overall) and changes in scores between weeks 1 and 5 were calculated. As data were not numeric, a non-parametric Wilcoxon signed rank test was then conducted to compare the change from baseline scores between treatments.

## 3 Results

### 3.1 Trial A: Bowel habit analysis

**Fig. 3:**
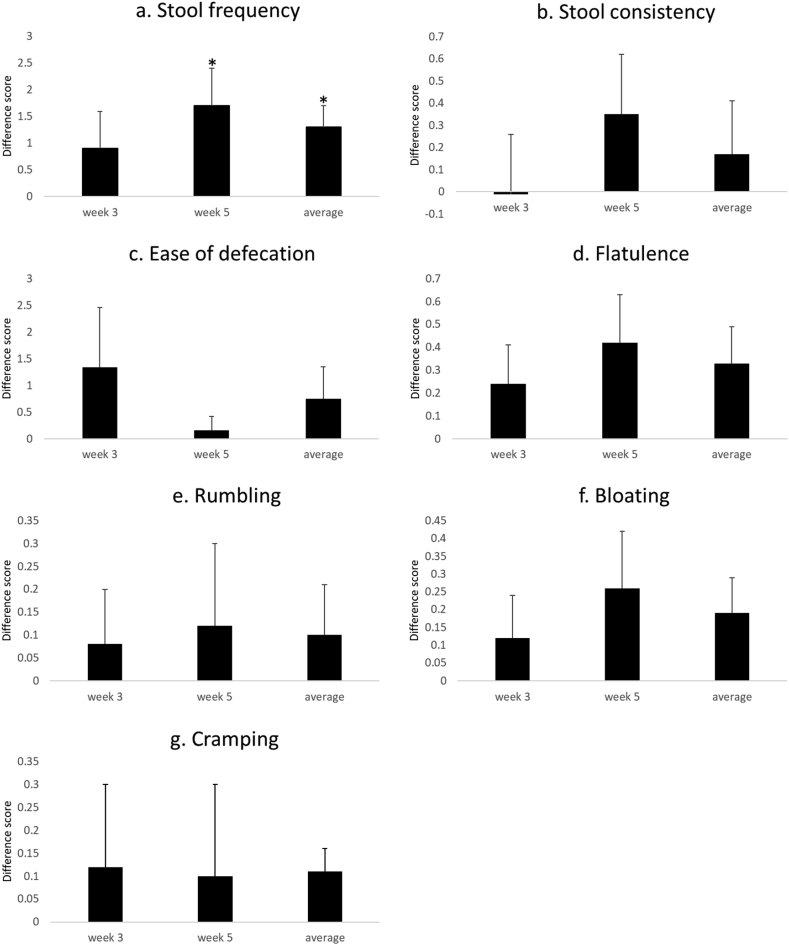
Mean difference scores, standard errors for all bowel habit data from Trial A. For each subject and outcome the difference score is the average value per day in the inulin period minus the average value per day in the placebo period. Data shown is the average difference score for weeks 3 and 5 and the combined difference scores for weeks 3 and 5 (average for the study). * denotes T-test outcomes for the values being different from 0.

The results of the bowel habit analysis for Trial A are presented in Fig. 3 and Supplementary Table 1. There was a significant increase in stool frequency at week 5 (*p* = 0.04) and for the average trial score (*p* = 0.01). These changes correspond to an increase in frequency of 1.3 bowel movements per week; thus bowel movements increased from an average ± standard error of mean (SEM) of 3.6 ± 0.25 to 4.9 ± 0.23 per week (Fig. 3a). No change in stool consistency was observed. There was a non-significant trend for an increase in bloating when weeks 3 and 5 were averaged *p* = 0.08), for flatulence at week 5 (*p* = 0.07) and averaged (*p* = 0.06), and for abdominal cramping for the average trial score (*p* = 0.06). Data tables can be found in Supplementary Table 1.

### 3.2 Trial B: Bowel habit analysis

**Fig. 4:**
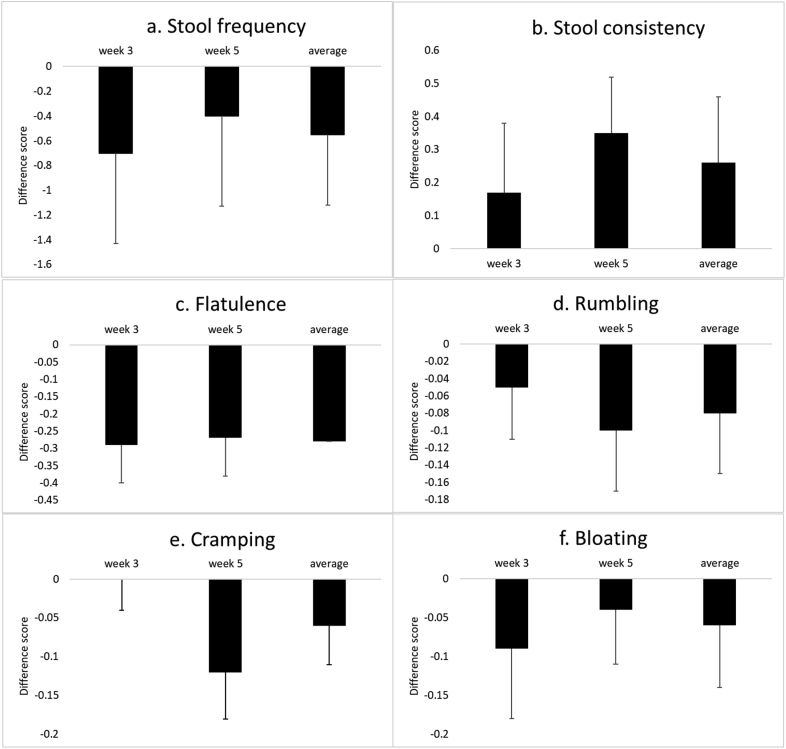
Mean difference scores and standard errors for all bowel habit data from the Trial B. Data shown is the average difference in score for weeks 3 and 5 and the combined difference scores for weeks 3 and 5 (trial average).

In Trial B there was no effect on stool frequency outcome when calculated as average of all volunteers (Fig. 4 and Supplementary Table 2). However, it was observed that the recorded number of stools per day was substantially higher than the inclusion criteria specified (i.e. two days or more per week without successful bowel movements). Across all weeks and treatments, including the first week of the placebo treatment, the average number was slightly above one bowel movement per day. In the first week of the placebo treatment, only six subjects (of the 20 that fully completed the bowel habit diary) had two or more days without any bowel movements per week on average in line with the inclusion criterion. It is noteworthy that the average difference scores during both weeks 3 and 5 for these six low-frequency subjects showed a non-significant increase in frequency of 0.28 per week from 5.1 to 5.4 bowel movements per week. The average frequency among subjects with high initial frequency was slightly reduced, by 0.89 per week, from 9.4 to 8.5 bowel movements per week, and this was also non-significant (*p* = 0.36)

The recorded values for both primary and secondary outcomes are shown in Supplementary Table 2. There were no overall changes in the stool frequency, and for the secondary outcomes, when the alpha level is corrected for multiple comparisons (Bonferroni, α = 0.008), none of the results reached statistical significance within the trial.

### 3.3 Response of initial low stool frequency subjects to inulin (both trials)

Interactions between inulin versus placebo treatment with low or high initial stool frequency in subjects from Trials A and B were analysed for those outcomes in the bowel habit diaries or questionnaires where identical scales were used. The number of volunteers included in the combination of trials was 30. Histograms produced in the Minitab output were visually analysed and visible outliers were removed from the analysis. Analyses reported in this section are data with outliers omitted and the number of volunteers included in each analysis along with analysis outcomes can be found in Table 1.

**Table 1:** Mean difference scores and standard errors for bowel habit data from all subjects in Trials A and B, and subjects with low initial stool frequency (LISF) from Trials A and B (A & B-LISF). Data shown as ‘Mean’ are the average of difference scores for weeks 3 and 5, with corresponding SEM.

Outcome	Data from Trial	Number of subjects	Mean	SEM	*p*-value -interaction LISF versus HISF‡	P-value
Stool frequency	A & B	28	0.08	0.05	0.025*	0.98
	A & B-LISF only	16	0.91	0.06		0.032*§
Stool consistency	A & B	29	0.28	0.10	0.91	0.008*
Flatulence	A & B	30	−0.14	0.09	0.39	0.125
Rumbling	A & B	30	−0.02	0.05	0.43	0.67
Bloating	A & B	29	0.03	0.04	0.125	0.39
Cramping	A & B	30	−0.02	0.03	0.088	0.67

*denotes significant difference <0.008 (for secondary outcomes with Bonferroni correction).

† *p*-values for ‘Trials A & B’ indicate if the difference scores for all subjects together are different from 0.

‡ *p*-value interaction LISF versus High Initial Stool Frequency (HISF) show if the subjects in both trials with LISF had a significantly different outcome than those in Trial B subjects with HISF. § *p*-value for stool frequency, where this interaction is significant for subjects in Trial A & B-LISF, shows the difference between inulin treatment and placebo was significant for those subjects in both trials who had low initial frequency.

When the trial data for stool frequency for all subjects in Trial A and B were combined, stool frequency was not significantly different between inulin and placebo treatments. However, there was an interaction with initial frequency of defecation (*p* = 0.025), and the low frequency subjects showed an increase in stool frequency of 0.7 per week compared to placebo. In addition, there was an increase in stool type implying softer stool consistency of 0.28 points on the Bristol stool chart when data of both trials were combined, irrespective of initial stool frequency (*p* = 0.008). When the alpha level for this secondary outcome was corrected for multiple comparisons (Bonferroni, α = 0.008), this was significantly different. There was a non-significant increase in cramping in subjects with a low initial frequency from both Trials A and B (*p* = 0.08). There were no other significant differences for subjects in Trial B with either low or high initial stool frequency (data not shown).

### 3.4 Assessment of constipation symptoms in trial B

The outcome from the PAC-SYM questionnaire showed that no significant differences were found between inulin or placebo consumption for changes from the start to the end of each treatment period of any of the symptoms of discomfort tested using this validated questionnaire (Table 2). Global (overall) symptoms were reduced by a median of 2.5 points compared to the placebo (i.e. −4.5 compared with −2), however, all symptoms improved during the 5-week period irrespective of the treatment (all change values were negative except rectal); this suggests a strong placebo effect. Still, the reduction of global symptoms by a median value of 4.5 points in the subjects on inulin treatment strongly indicates a lack of significant side effects due to inulin.

**Table 2:** Median change from baseline score and score range after 5 weeks of treatment and outcomes of the Wilcoxon Rank Test for all PAC-SYM variables (symptoms).

Symptoms	Treatment	Median	Range	P- Value
Abdominal	Inulin	−0.5	2–−6	0.29
	Placebo	−1	6–−4	
Stool	Inulin	−3	4–−11	0.54
	Placebo	−2	4–−10	
Rectal	Inulin	0	3–−5	0.72
	Placebo	0	3–−4	
Global	Inulin	−4.5	4–−22	0.76
	Placebo	−2	−10–−12	

### 3.5 Impact of inulin on faecal microbiota in trial B

**Fig. 5:**
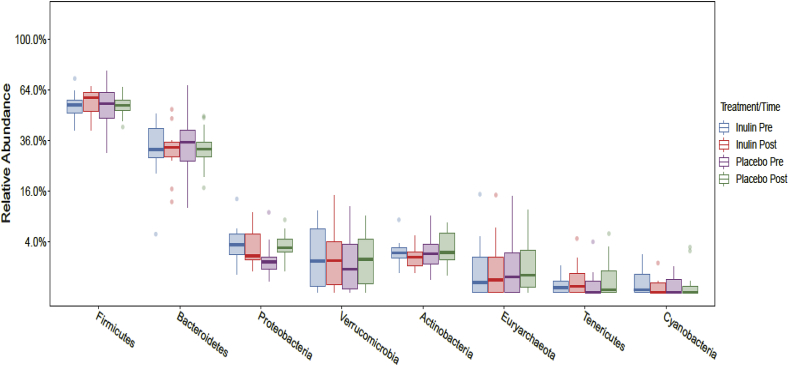
Boxplots showing the relative abundance of the top eight bacterial phyla prior to and following 5 weeks consuming inulin or placebo. No significant differences were identified.

**Fig. 6:**
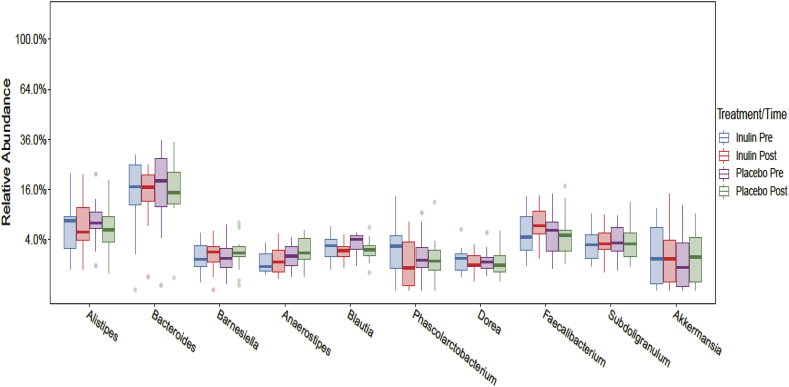
Boxplots showing the relative abundance of the top ten bacterial phyla prior to and following 5 weeks consuming inulin or placebo. No significant differences were identified.

**Fig. 7:**
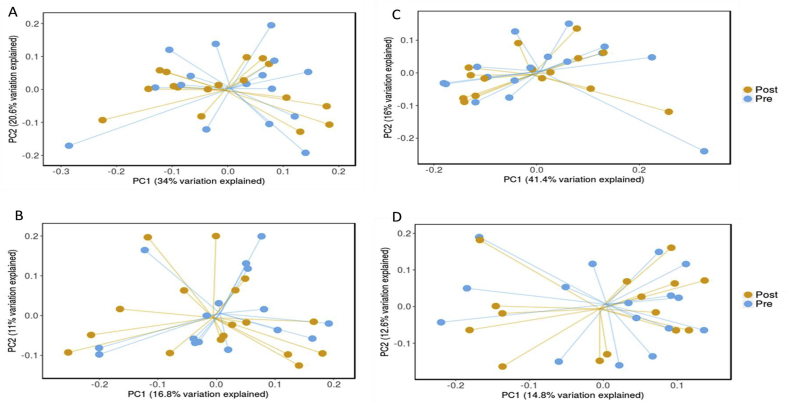
Principle component analysis of Weighted and Unweighted Unifrac analysis for pre and post Placebo (A and B, respectively) and Inulin (C and D, respectively).

Following a rarefaction curve analysis 4 samples were removed from the Inulin period (2 pre and 2 post). For these volunteers all remaining samples were removed from Inulin and Placebo arms periods of the study, giving a final sample size of 20 for Inulin and Placebo. Compositional analysis showed that Bacteroidetes and Firmicutes were the dominant phyla at baseline in the stool microbiota and following both arms of the intervention, with no significant differences (*p* > 0.05) between the inulin and placebo at pre or post each arm of the intervention. Volunteers had a mean of 33% and 32% for Bacteroidetes, and 55% and 56% for Firmicutes prior to consuming the placebo or inulin, respectively (Fig. 5). At *genus* level in both the placebo and inulin periods of the study, the dominant bacteria were *Bacteroides, Alistipes, Faecalibacterium and Subdoligranulum* (Fig. 6). Following the 5 week intervention there was a marginal increase in *Alistipes (p* = 0.61) *and Bifidobacterium* (*p* = 0.33), which were not present in the placebo arm of the study, and a larger increase in *Faecalibacterium (p* = 0.61), following inulin consumption (Fig. 7 and Supplementary Table 3). Following 5 weeks intervention, there were no significant changes at phylum or *genus* level (*p* > 0.05) in either the placebo or inulin consumption arm of the study. We also compared the differences between pre and post inulin and placebo periods in the change from pre to post (effect size), however, none of the effects were significant (all *p* > 0.05; Supplementary Table 3).

α-Diversity was analysed by comparing the number of OTUs and Shannon Index prior to and following each individual arm of the study. α-Diversity analysis demonstrated that there was no significant difference between diversity of samples collected at baseline and following 5 weeks of the intervention in the placebo arm of the study (*p* = 0.73). There was a small increase in the number of OTUs following the inulin intervention (*p* = 0.42), which was mirrored by a small decline in OTUs following placebo consumption, suggesting inulin supplementation may be responsible for this increase. (Supplementary Fig. 1 and Supplementary Table 3). The Shannon diversity index, a measure of richness and evenness (i.e., how evenly an OTU is distributed throughout the sample) was not significantly different between pre and post for the placebo (*p* = 0.53) or inulin (*p* = 0.25) arms of the trial (Supplementary Table 3). There was no significant difference in the changes of OTUs and Shannon Index between the inulin and placebo periods (all P > 0.05; Supplementary Table 3).

To assess β-diversity, weighted and unweighted Unifrac were conducted and visualised using principle co-ordinate analysis (PCoA). There was no significant difference in β-diversity between pre and post placebo demonstrated by the Weighted Unifrac (*p* = 0.95, R-squared = 0.01) and Unweighted Unifrac (*p* = 1.00, R squared = 0.01) (Fig. 7 A and B, respectively). There was also no significant difference in β-diversity between pre and post following inulin supplementation, Weighted Unifrac (*p* = 0.85, R squared = 0.02), Unweighted Unifrac (*p* = 0.99, R squared = 0.01) (Fig. 7 C and D, respectively) and Bray Curtis (*p* = 0.97, R squared = 0.02).

We investigated the impact of initial stool frequency on gut microbiota composition, as determined using the inclusion criteria; low frequency: ‘≥ two days without bowel movements per week during week 1 of the placebo period', and high frequency: '< two days without bowel movements'. There were no significant changes in any taxa abundance or overall gut microbiota composition between low and high stool frequency volunteers within or between the inulin and placebo periods prior to or following the five week interventions (*p* > 0.05).

Combining the sequence data from all volunteers at all time points we found significant associations between volunteers (*p* = 0.004) and gender (*p* = 0.006) with microbiota profiles, accounting for 87% and 11% of the overall variability, respectively. Time point and treatment period were not significantly associated with the microbiota profiles (*p* = 0.920) and (*p* = 0.922), respectively, explaining only 0.3% and 0.1% of the overall variability, respectively. This corroborates the previous findings that each volunteer has a unique bacterial community, which is stable over time despite inulin intervention.

## 4 Discussion

These randomised, double blind, placebo-controlled trials assessed the effects of inulin on bowel symptoms of constipation, with stool frequency as the primary outcome, quality of life and gut microbiota composition in middle-aged to elderly populations. The study demonstrated that 10  g/d of inulin improved stool frequency and consistency in older adults only with low stool frequency; there were no other significant changes in gastrointestinal symptoms. There were no significant changes in gut microbiota composition.

In order to participate in this study, subjects were screened for low stool frequency with the criterion for inclusion set at two to four days per week without successful bowel movement (since they were excluded if they had clinically relevant constipation, defined according to the Rome criteria as less than three successful bowel movements per week (Longstreth et al., 2006). This criterion was challenging to achieve. In Trial A, there was a significant increase of greater than 1 additional bowel movement per week in stool frequency between the subjects consuming inulin versus the placebo; all these subjects had low initial stool frequency. In Trial B, there was no overall effect of the inulin on stool frequency, however, many subjects in Trial B did not report a low stool frequency during the trial, notably also not for the placebo. The recorded number of stools per day for the Trial B subjects was substantially higher in the first week of the trial, in fact the average exceeded one bowel movement per day, irrespective of treatment. There are several possible explanations for this. One is a possible strong placebo effect from participating in this study (Staudacher, Irving, Lomer, & Whelan, 2017). In addition, some volunteers had very variable bowel movements during Trial B, with multiple stools on some days and none on others. This may create an impression to the subject of frequently being unable to pass stools, even though the total frequency of successful attempts was not particularly low. Prolonged screening of this parameter for 2–3 weeks prior to commencing the trial is recommended for future studies, preferably with all volunteers receiving a placebo product during this stage.

Interestingly, when data of subjects with low initial stool frequency from Trials A and B were combined, there was a statistically significant increase in frequency of 0.77 defecations per week. These improvements in defecation frequency are in line with previous reports on subjects with more serious clinically relevant constipation (Den Hond et al., 2000; Micka, Siepelmeyer, Holz, Theis, & Schön, 2017; Sobotka et al., 1997). It appears that the effect of native inulin to improving stool frequency can be effectively observed in persons with low stool frequency without a clinical diagnosis of constipation.

In contrast, for the 12 subjects with high initial stool frequency, inulin consumption appeared to result in a (non-significant) reduction (−0.89 ± 0.75) in the frequency of defecation. This may be explained if volunteers pass fewer larger stools rather than several smaller ones, an outcome which has been previously shown upon inulin consumption (Castiglia-Delavaud et al., 1998; Causey, Feirtag, Gallaher, Tungland, & Slavin, 2000). In future studies this may be monitored using measurements of faecal output to assess if stool size is increased following inulin consumption. Irrespective of the reasons for the high initial frequency in some volunteers, we report that following inulin consumption stool frequency was marginally increased. These data suggest that volunteers with variable stool frequency may benefit from inulin in terms of improving regularity. However the present study was not designed or powered to test for such an effect.

The role of the gut microbiota in patients with poor bowel movements has received considerable interest over the last decade, which has predominantly been possible due to technological advances. Irrespective of the exact aetiology, altered intestinal transit is associated with altered bacterial composition and gut microbiota dysbiosis (VAN Felius et al., 2003). Inulin has been shown to improve bowel movements, GI symptoms, quality of life and increase faecal output in various adult populations (Castiglia-Delavaud et al., 1998; Den Hond et al., 2000; Marteau et al., 2011; Vandeputte et al., 2017a). These improvements have generally been associated with modest changes in gut microbiota composition, specifically *Bifidobacteria*, but also with *Bilophila, Anaerostipes and Clostridium* in constipated subjects amongst others (Dewulf et al., 2013; Kim, Lee, & Meyer, 2007; Linetzky Waitzberg et al., 2012; Vandeputte et al., 2017a). These changes are likely due to the ability of inulin to reach the colon where it is fermented by bacteria, producing short chain fatty acids, which are recognised as health promoting metabolites (Kau, Ahern, Griffin, Goodman, & Gordon, 2011).

In the current study we observed an increase in stool frequency in volunteers with low stool frequency, as previously reported (Vandeputte et al., 2017a). This could ultimately affect the delivery of nutrients to the gut microbiota, influence luminal pH and environmental conditions. In contrast to previous studies (Schaafsma & Slavin, 2015; Vandeputte et al., 2017a), we did not report any significant changes in specific bacterial taxa, notably *Bifidobacterium*, or changes in alpha or beta diversity. This outcome may be due to the small number of subjects, population selected, geographical location, inclusion/exclusion criteria and/or other aspects of the study design. There is evidence in animal studies to show that the response to inulin may be dependent upon the gut microbiota composition at study commencement (Rodriguez et al., 2018). Here we reported non-significant increases in *Faecalibacterium, Bifidobacterium and Alistipes*, all of which are associated with gut health, including increased bacterial diversity, reduced triglycerides (Zhernakova et al., 2016), metabolic control and short chain fatty acid production (Varela et al., 2013; Xu et al., 2015). In contrast to previously published studies (Dewulf et al., 2013; Kim et al., 2007; Linetzky Waitzberg et al., 2012; Vandeputte et al., 2017a) we did not report any significant changes in gut microbiota composition, despite the improvements in stool frequency. The present study may not have been sufficiently powered to detect these differences, in particular considering the effect of the initial gut microbiota composition as previously suggested (De Preter et al., 2008; Bouhnik et al., 2004). For example De Preter et al. (2008) reported that the relative increase in *Bifidobacteria* were dependent on the initial concentrations of *Bifidobacteria* following inulin consumption in healthy volunteers. However from the perspective of contributing data to future meta-analyses on this subject, it is noticeable that the directions of the observed trends generally correspond well with the significant effects in other studies. This is also the reason why the non-significant trends for other outcomes are reported in the present paper.

The only significant secondary outcome of the trial was the impact of 10 g of native inulin per day on stool consistency in the subjects. Results from Trial A and Trial B separately showed no statistically significant change in stool consistency. However, when data from both trial sites were combined, stool consistency increased by 0.29 points on the Bristol stool chart (*p* = 0.008, after correction for multiple outcomes) a finding also reported after consuming 12 g of Orafti^®^ inulin per day for 4 weeks (Micka et al., 2017).

We conclude that 10 g native chicory inulin improved stool frequency and stool consistency in otherwise healthy middle-aged adults with unsatisfactory low stool frequency. Inulin consumption did not modulate taxa abundance or overall gut microbiota composition significantly, however the directions of observed trends were consistent with published data on other types of inulin. Importantly there were no indications of potentially negative effects on gastrointestinal symptoms in these subjects.

## Clinical trial registration

Trial A was registered at CCMO (http://www.ccmo.nl/en/ccmo-register) with registration number NL27269.003.09 and Trial B was registered at DOI 10.1186/ISRCTN97558933.

## Appendix A Supplementary data

Supplementary Documents_V2 Supplementary Documents_V2

Supplementary Document 2 Randomisation procedure macro read only.xlsx Supplementary Document 2 Randomisation procedure macro read only.xlsx
