# Supplementary material for: Changes in stool frequency following chicory inulin consumption, and effects on stool consistency, quality of life and composition of gut microbiota
Source: Food Hydrocoll. 2019 Nov;96:688–98. doi: 10.1016/j.foodhyd.2019.06.006 (PMC6686634; doi:10.1016/j.foodhyd.2019.06.006)
Supplement: Supplementary Documents_V2 [file mmc1.docx]

**ONLINE SUPPLEMENTARY MATERIALS**

**Supplementary Document 1**

**Randomization and blinding algorithm**

Two randomization lists were prepared, one for females and one for males. Volunteers were assigned to groups according to the next available item on the list. In order to minimise the risk of unbalanced allocation of groups within a gender if the number of participants of this gender is low, the randomization list sequences were sequential with a base number of two and with 20% random offset. This means that for each two consecutive participants, the first one was randomly assigned to one treatment group, the subsequent volunteer was then assigned to the other group. The offset (in order to disguise the sequence) was that an additional volunteer was inserted at random before 20% of these pairs. The offset positions were randomly allocated to group independent of the sequence.

An Excel file was developed with a macro that automatically generates a new set of randomisation sequences every time the file is opened (Supplementary Document 2). Each set of codes consists of one list of volunteer allocation codes, specifying which volunteer will receive packages labelled with a particular labelling code (1, 2, 3, 4, 5 and 6), plus a separate list of labelling codes to mark the packages of sachets, specifying labelling codes to use for inulin packages and codes to use for placebo packages (A1, A2, A3, A4, A5, A6, B1, B2, B3, B4, B5 and B6; A for the first period, B for the second). The file generates codes for up to 33 volunteers in each gender. If this is not sufficient, a new set of 33 allocations can be produced.

This file was sent to Sensus BV. For each gender, the file was opened or recalculated and the generated sequence (two pages) printed as the master sequence for this gender. The original master sequences for males and females were used to produce two sets of opaque sealed envelopes, one for each gender, each marked on the outside with the gender and a volunteer number (1-33) and inside containing the labelling code (1-6). The envelopes were sent to Newcastle University, which opened the next envelope when a volunteer was enrolled following the screening visit. Once these sealed envelopes was sent to Newcastle, and before the first volunteer was enrolled, the master copy of these lists was placed in another sealed envelope and kept by Sensus BV in a safe place, in case it would become necessary to check the master copy.

Regarding the product labelling allocation, another two sets of sealed opaque envelopes were marked on the outside with the gender and labelling code (1-6) and on the inside with the treatment in periods A and B (inulin or placebo). This set of sealed envelopes were also sent to Newcastle University, however only as a safety precaution, they were only to be opened in case of an adverse event, where emergency unblinding was required. Sensus then kept the product labelling allocation codes available, without showing them to Newcastle University staff or students. In this way it was able to prepare pre-labelled product packages for each gender with the labelling codes (1A-6B) and send the labelled packages to Newcastle as required.

**Assessment of suitability of volunteers**

The phrase *‘any other condition that in the view of the volunteer’s general practitioner or the study manager may make the volunteer unsuitable for the trial’* covers a wide range of conditions, from pregnancy and migraine to various physical or mental issues not directly related to the outcomes of the trial. They have in common that after individual assessment they may lead to exclusion, in cases where the potential disadvantages for the volunteer and/or the outcomes may on balance exceed the advantages. In the present trial, this provision was not actually used to exclude any potential volunteers. However, for one of the volunteers, their general practitioner was consulted to confirm that the volunteer would be fit to participate in the trial despite receiving medication for a well-controlled mental condition.

**Examples of products containing inulin or probiotics.**

Provided to volunteers in trial B to indicate for which types of products they should carefully check the ingredients list and avoid if they were found to contain inulin or probiotics.

A similar list (in Dutch) was provided to volunteers in trial A.

Examples of Cakes, Pastries & Sweet Goods


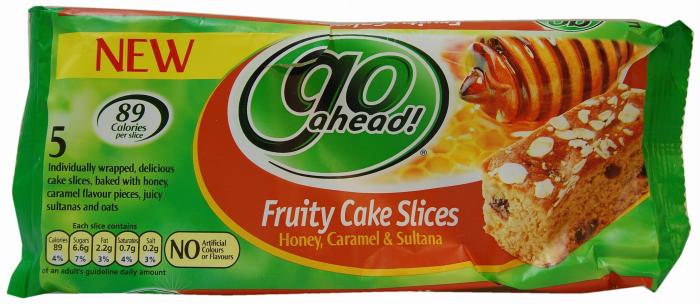

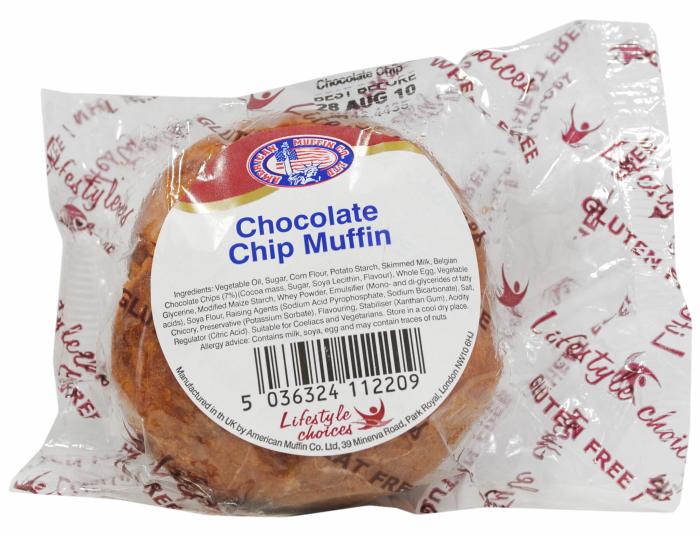


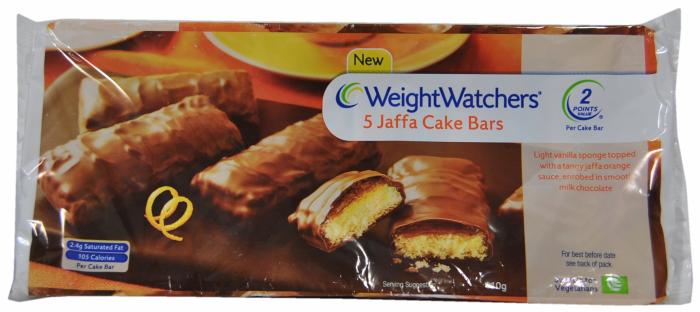

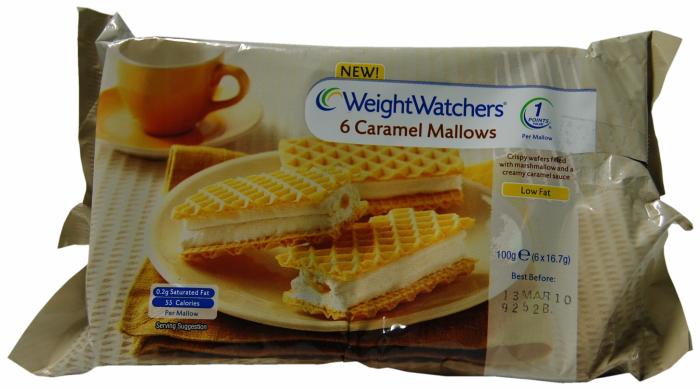


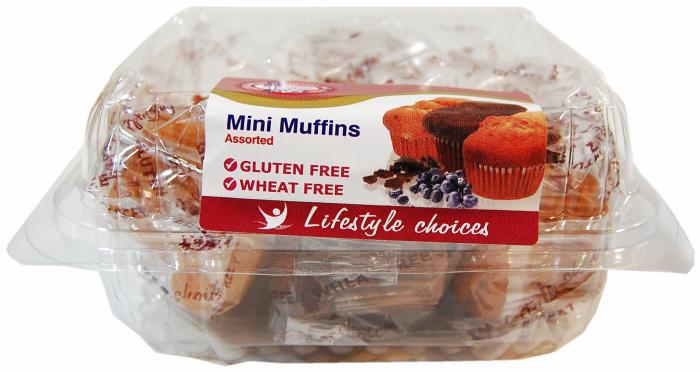

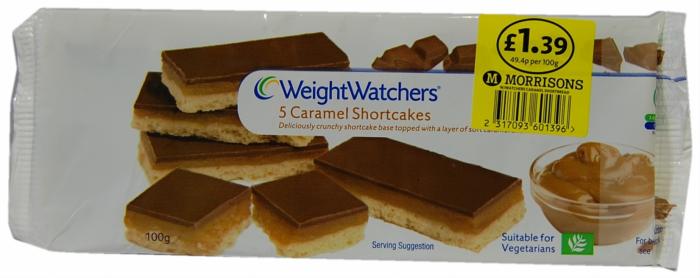


Example of cookies:


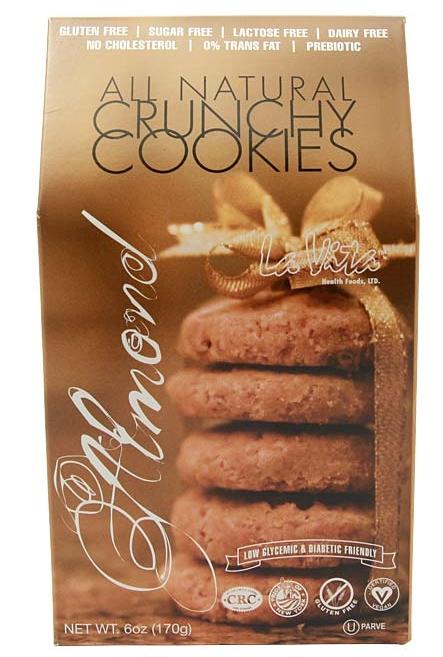


Examples of dairy products: Drinking Yogurts, Yogurts and Liquid Cultured Milk


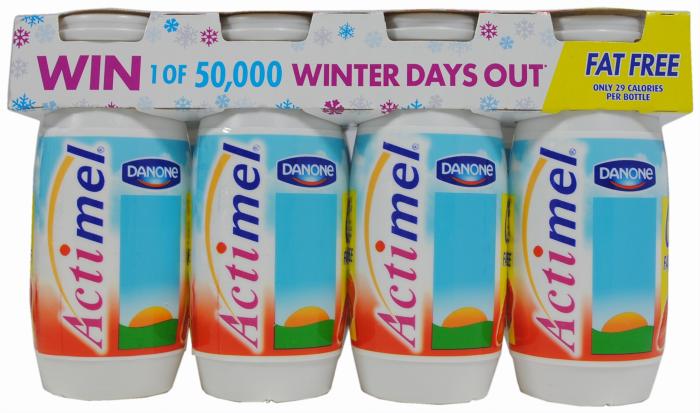

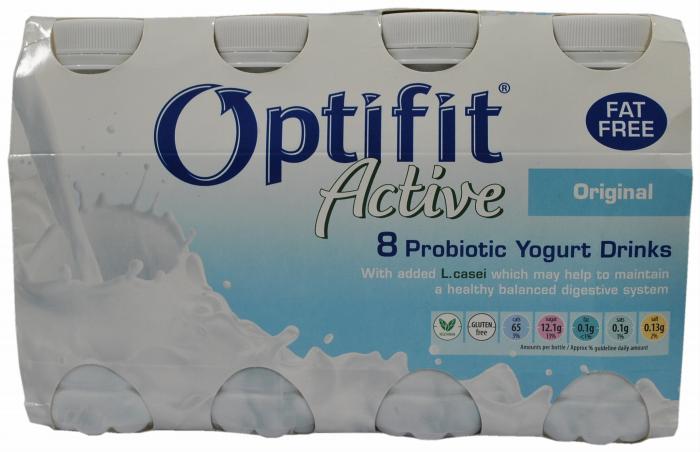


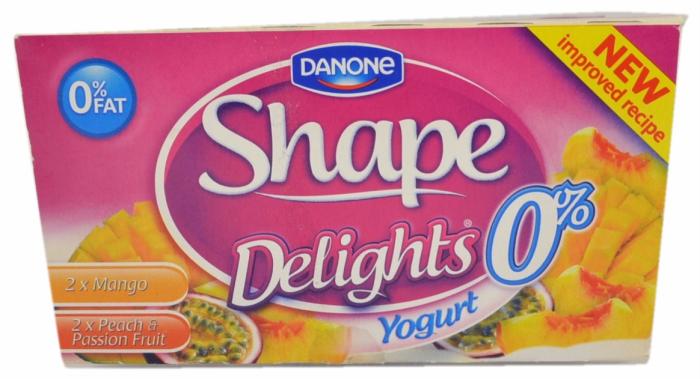

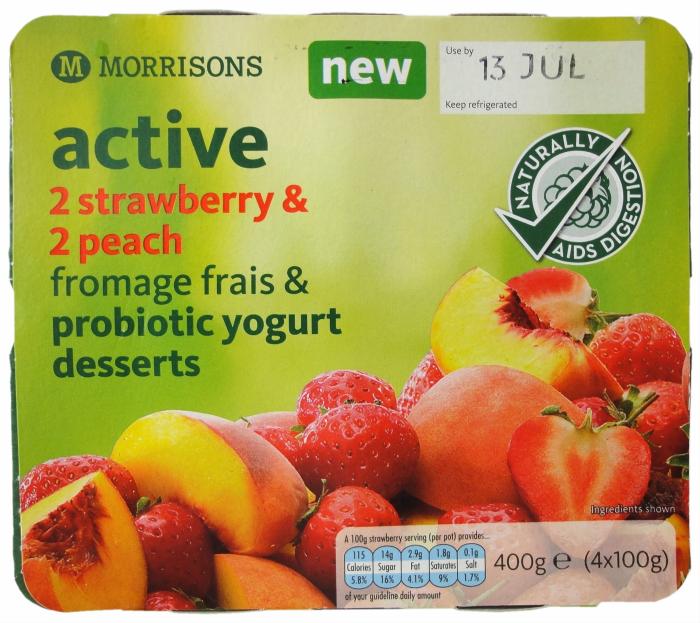


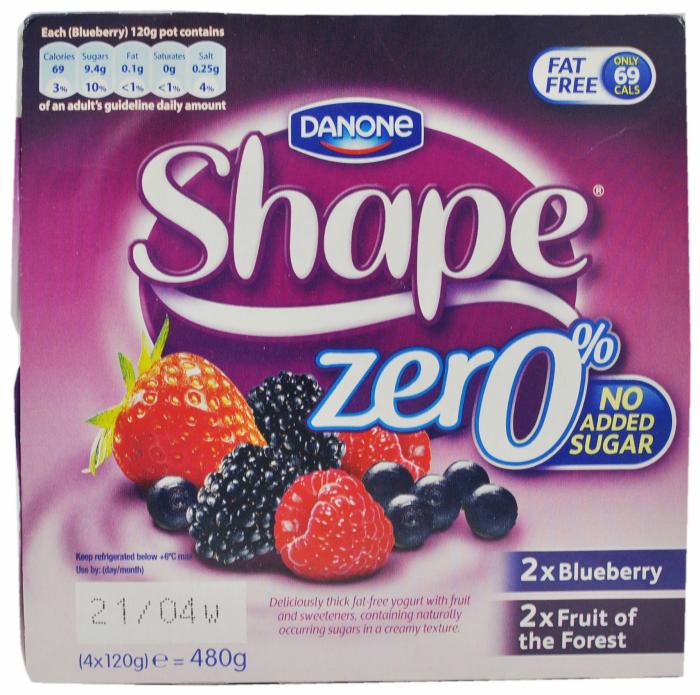

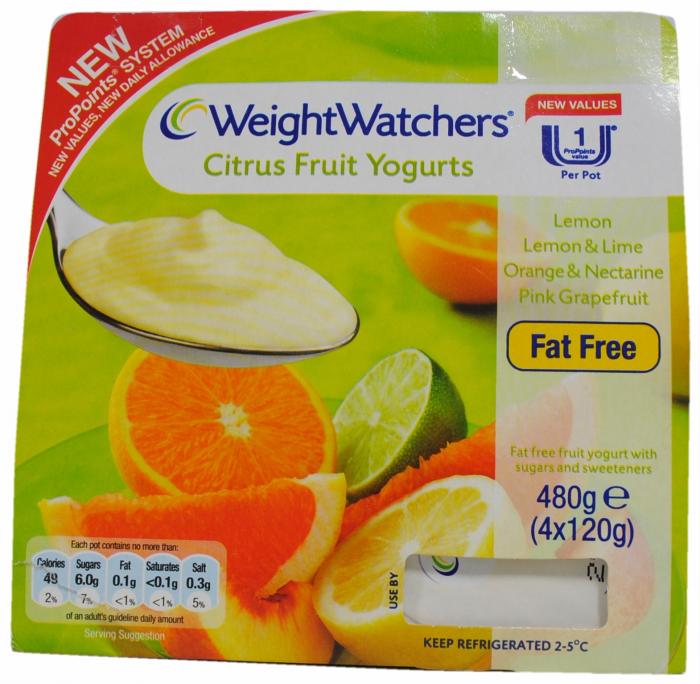


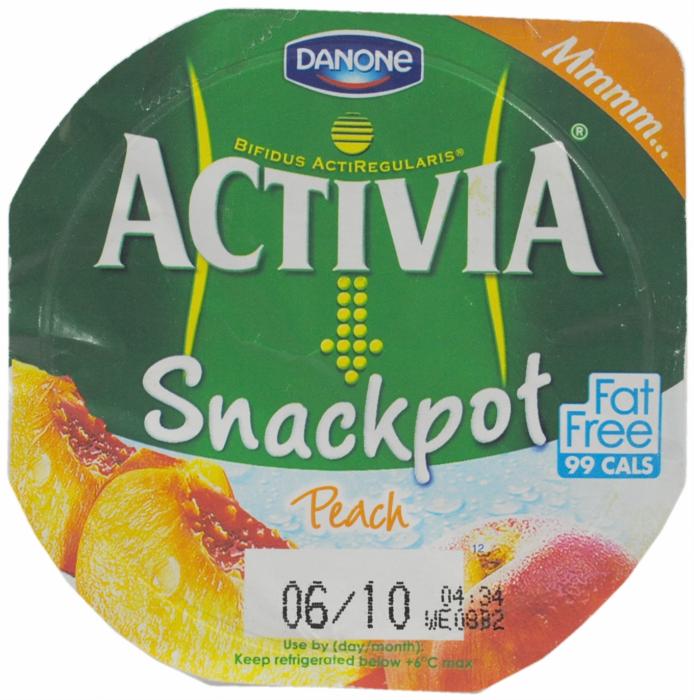


Examples of breakfast cereals:


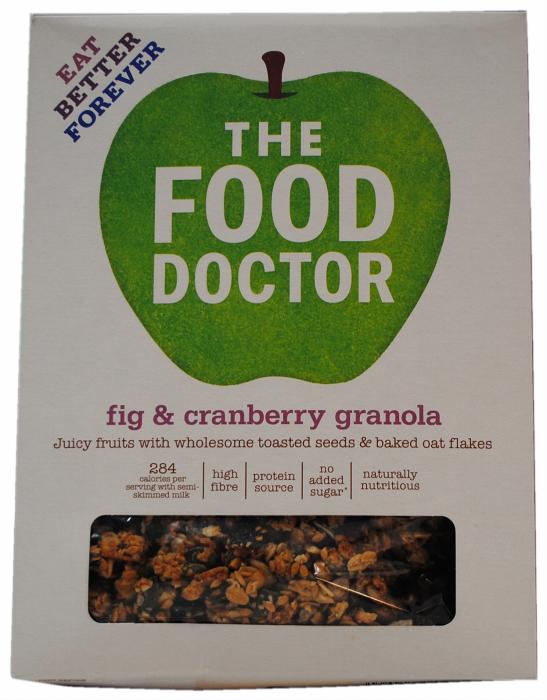

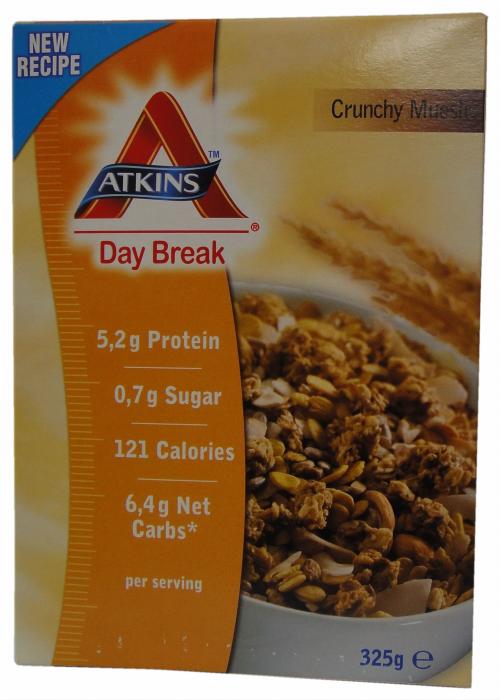

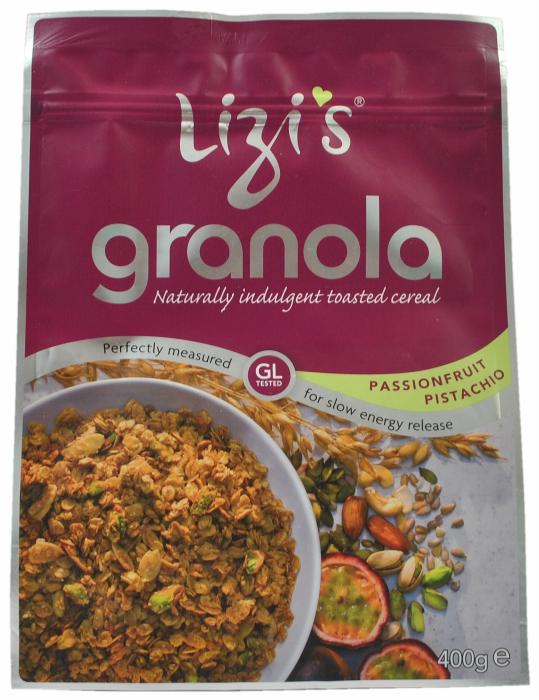


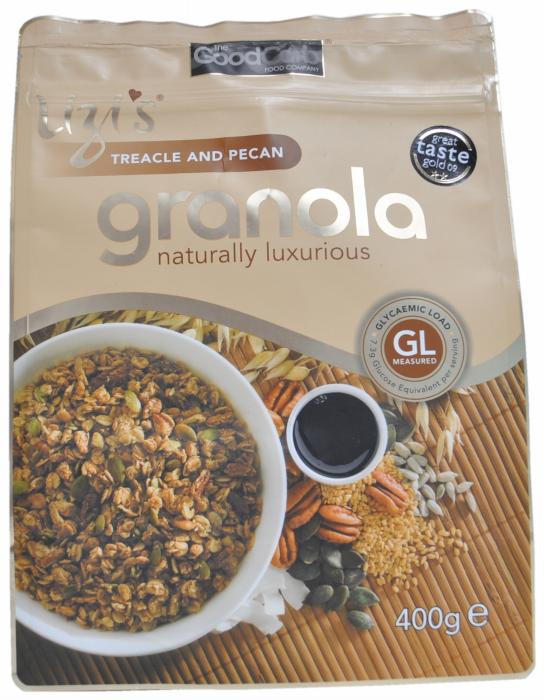

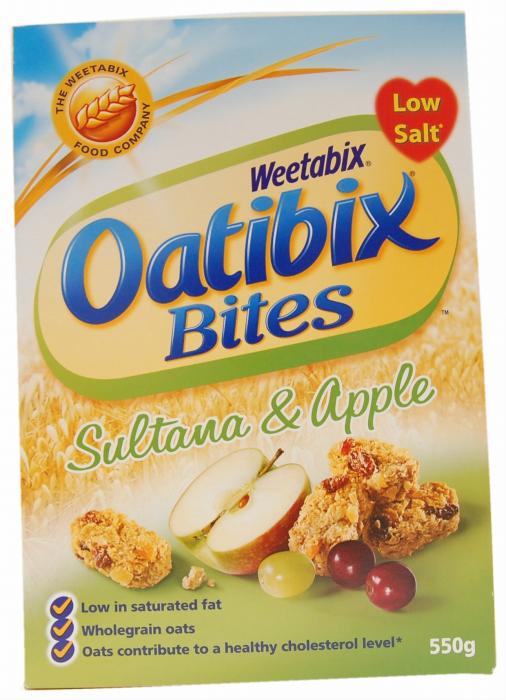

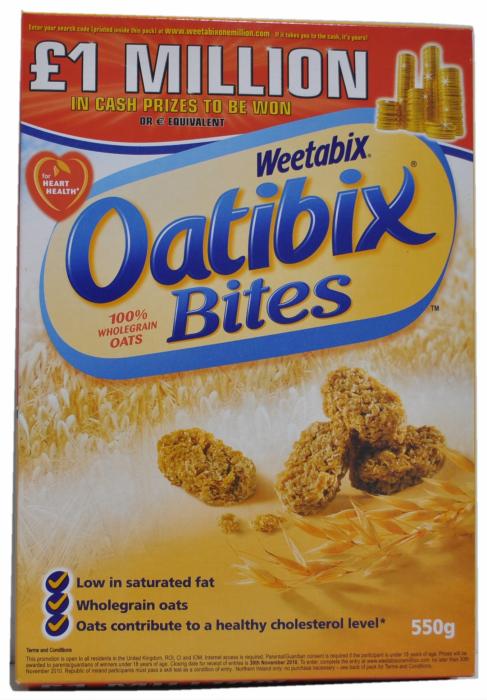


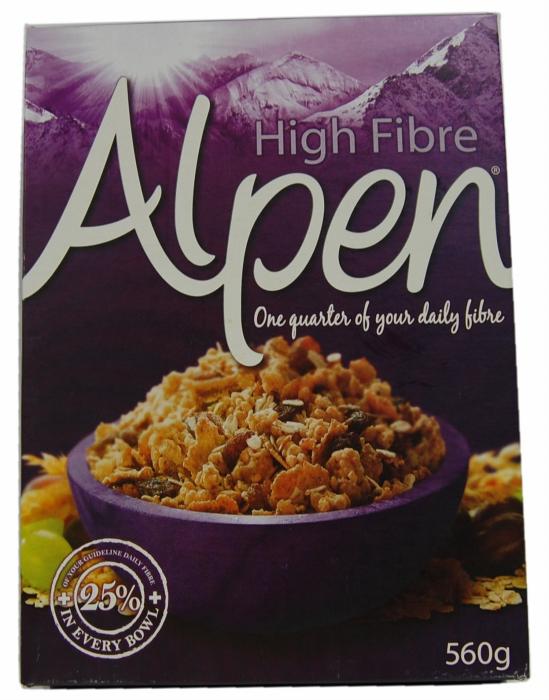


Examples of snack/cereal/Energy bars


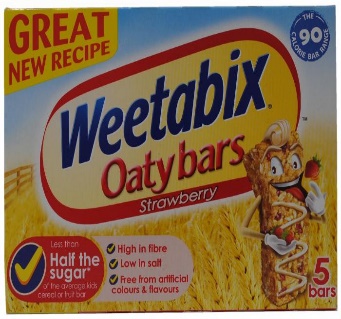

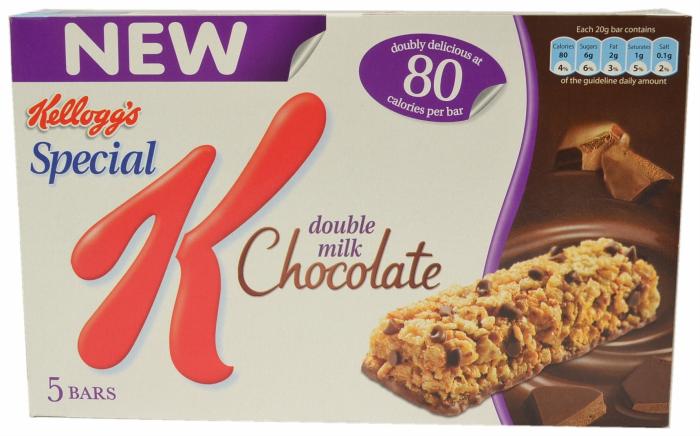

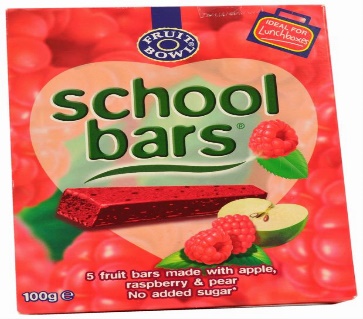

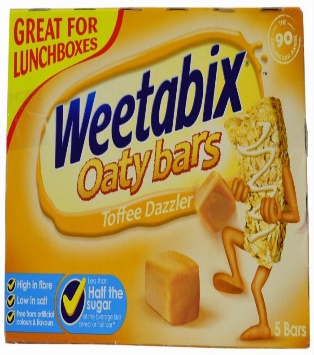

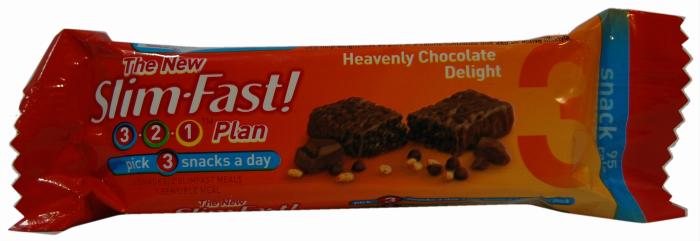

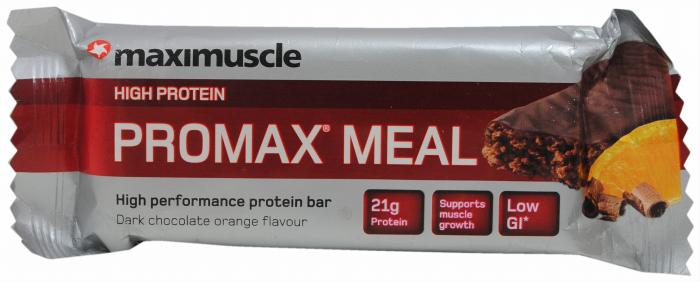


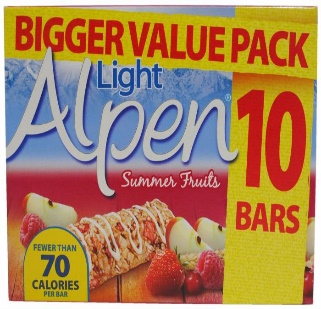

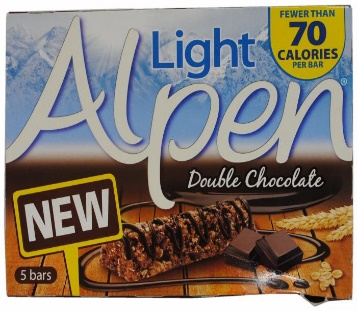

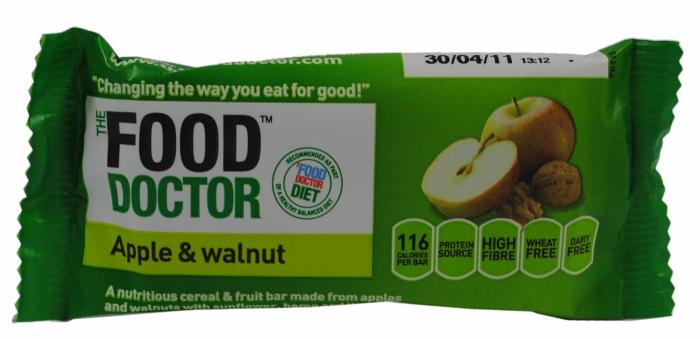


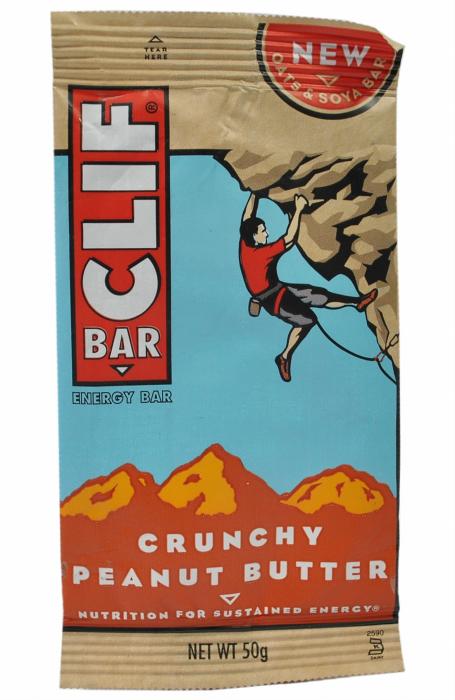

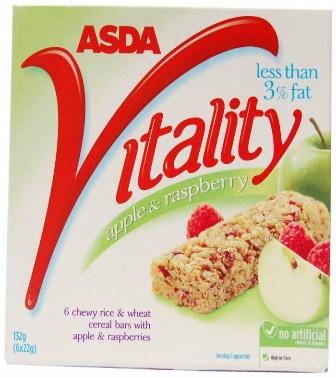


Example of bread products:


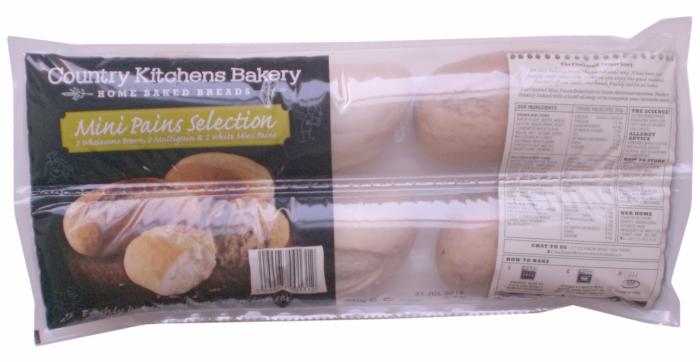


**Supplementary Table 1.** Mean difference scores, standard errors and T-test outcomes for the values being different from 0, for all bowel habit data from Trial A. For each subject and outcome the difference score is the average value per day in the inulin period minus the average value per day in the placebo period. Data shown is the average difference score for weeks 3 and 5 and the combined difference scores for weeks 3 and 5 (average for the study).

| Outcome per week / average | | Mean | | SEM | | T-test value | | *P* value | |  |
| --- | --- | --- | --- | --- | --- | --- | --- | --- | --- | --- |
| Stool frequency | week 3 | | 0.90 | | 0.69 | | 1.30 | | 0.22 | |
|  | week 5 | | 1.70 | | 0.70 | | 2.43 | | 0.04* | |
|  | average | | 1.30 | | 0.40 | | 3.23 | | 0.01* | |
| Stool consistency | week 3 | | -0.01 | | 0.27 | | -0.04 | | 0.97 | |
|  | week 5 | | 0.35 | | 0.27 | | 1.26 | | 0.24 | |
|  | average | | 0.17 | | 0.24 | | 0.71 | | 0.50 | |
| Defecation ease | week 3 | | 1.34 | | 1.12 | | 1.19 | | 0.26 | |
|  | week 5 | | 0.16 | | 0.26 | | 0.61 | | 0.56 | |
|  | average | | 0.75 | | 0.60 | | 1.24 | | 0.25 | |
| Flatulence | week 3 | | 0.24 | | 0.17 | | 1.43 | | 0.19 | |
|  | week 5 | | 0.42 | | 0.21 | | 2.02 | | 0.07 | |
|  | average | | 0.33 | | 0.16 | | 2.12 | | 0.06 | |
| Rumbling | week 3 | | 0.08 | | 0.12 | | 0.65 | | 0.53 | |
|  | week 5 | | 0.12 | | 0.18 | | 0.65 | | 0.53 | |
|  | average | | 0.10 | | 0.11 | | 0.93 | | 0.38 | |
| Bloating | week 3 | | 0.12 | | 0.12 | | 1.00 | | 0.34 | |
|  | week 5 | | 0.26 | | 0.16 | | 1.68 | | 0.13 | |
|  | average | | 0.19 | | 0.10 | | 1.98 | | 0.08 | |
| Cramping | week 3 | | 0.12 | | 0.18 | | 0.65 | | 0.53 | |
|  | week 5 | | 0.10 | | 0.20 | | 0.50 | | 0.63 | |
|  | average | | 0.11 | | 0.05 | | 2.18 | | 0.06 | |

* denotes significance at <0.05. A graphical representation of results can be found in Figure 3

**Supplementary table 2.** Mean difference scores, standard errors and T-test outcomes for the values being different from 0, for all bowel habit data from the Trial B. Data shown is the average difference in score for weeks 3 and 5 and the combined difference scores for weeks 3 and 5 (trial average).

| Symptom outcome per week / average | | Mean | SEM | T-test value | *p* value |
| --- | --- | --- | --- | --- | --- |
| Stool frequency | week 3 | -0.70 | 0.73 | -0.96 | 0.35 |
|  | week 5 | -0.40 | 0.73 | -0.55 | 0.59 |
|  | average | -0.55 | 0.57 | -0.96 | 0.35 |
| Stool consistency | week 3 | 0.17 | 0.21 | 0.81 | 0.43 |
|  | week 5 | 0.35 | 0.17 | 2.06 | 0.05 |
|  | average | 0.26 | 0.20 | 2.21 | 0.04 |
| Flatulence | week 3 | -0.29 | 0.11 | -2.57 | 0.02 |
|  | week 5 | -0.27 | 0.11 | -2.52 | 0.02 |
|  | average | -0.28 | 0.00 | -2.84 | 0.10 |
| Rumbling | week 3 | -0.05 | 0.06 | -0.77 | 0.45 |
|  | week 5 | -0.10 | 0.07 | -1.42 | 0.17 |
|  | average | -0.08 | 0.07 | -1.21 | 0.24 |
| Bloating | week 3 | -0.09 | 0.09 | -1.01 | 0.32 |
|  | week 5 | -0.04 | 0.07 | -0.48 | 0.63 |
|  | average | -0.06 | 0.08 | -1.15 | 0.27 |
| Cramping | week 3 | 0.00 | 0.04 | 0.00 | 1.00 |
|  | week 5 | -0.12 | 0.06 | -1.95 | 0.07 |
|  | average | -0.06 | 0.05 | -1.44 | 0.17 |

For the primary outcome Stool Frequency, a difference <0.05 would be significant. For the other outcomes, α=0.008 (due to Bonferroni correction). A graphical representation of results can be found Figure 3.

.


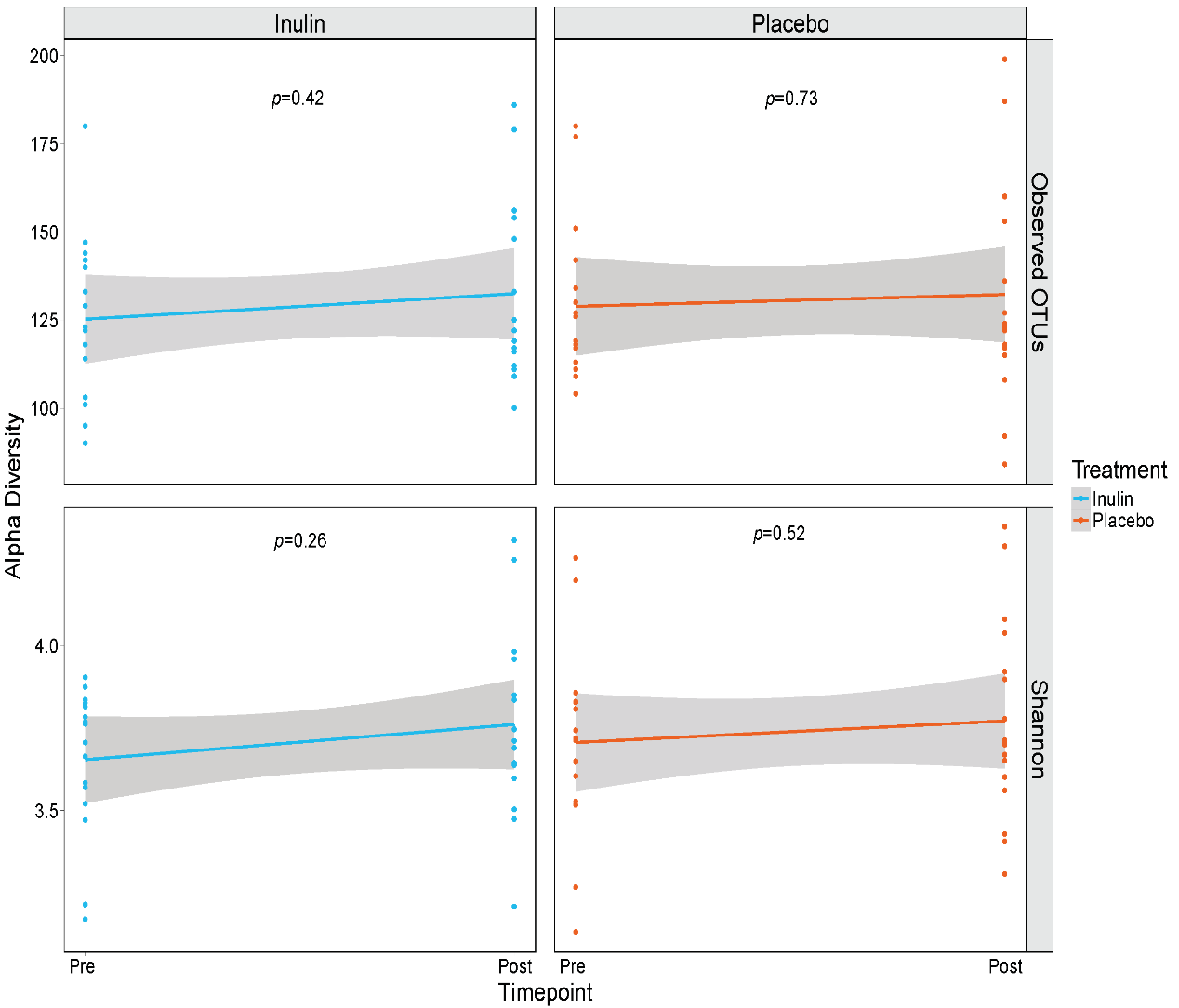


**Supplementary Figure 1:** Regression analysis showing number of observed OTU and Shannon Index differences between pre and post for inulin and placebo arms of the intervention. Grey shading denotes 95% confidence interval of the mean.

**Supplementary Table 3.** Mean (± SD) relative abundance of top eight most abundant phyla (bold), top eleven most abundant *genera* (italics) bacteria for Inulin and Placebo periods. Change (∆) between pre and post for Inulin and Placebo. Mean (± SD) differences between Inulin and Placebo ∆ and observed number of OTUs and Shannon Index pre and post inulin and placebo consumption.

|  |  | Inulin | | | |  | Placebo | | | |  | Inulin vs Placebo | |
| --- | --- | --- | --- | --- | --- | --- | --- | --- | --- | --- | --- | --- | --- |
|  |  | Pre (n=20) | Post (n=20) | ∆ Pre and Post | P Value* |  | Pre (n=20) | Post (n=20) | ∆ Pre and Post | P Value* |  | ∆ (n=20) | P Value^#^ |
| **Euryarchaeota** | | 95 (136) | 89 (110) | -1 (1) | 0.95 |  | 90 (112) | 73 (116) | 1 (47) | 0.70 |  | -13 (50) | 0.44 |
| **Actinobacteria** | | 86 (63) | 80 (52) | -8 (87) | 0.31 |  | 87 (60) | 91 (42) | 16 (63) | 0.58 |  | -28 (77) | 0.15 |
| *Bifidobactrium* | | 33 (25) | 43 (31) | 10 (25) | 0.33 |  | 64 (41) | 62 (52) | -2 (37) | 0.41 |  | 18 (61) | 0.18 |
| **Bacteroidetes** | | 1043 (320) | 1014 (254) | -162 (280) | 0.68 |  | 1048 (373) | 964 (284) | 71 (242) | 0.63 |  | -18 (275) | 0.90 |
| *Alistipes* |  | 205 (140) | 223 (158) | 14 (82) | 0.96 |  | 240 (153) | 227 (144) | -9 (99) | 0.58 |  | 15 (137) | 0.33 |
| *Bacteroides* |  | 502 (242) | 489 (178) | -19 (192) | 0.71 |  | 549 (268) | 540 (229) | -22 (201) | 0.75 |  | 7 (180) | 0.65 |
| *Barnesiella* |  | 56 (47) | 57 (42) | 8 (41) | 0.59 |  | 58 (57) | 69 (49) | 8 (36) | 0.42 |  | 1 (52) | 0.93 |
| **Cyanobacteria** | | 28 (24) | 11 (13) | -22 (35) | 0.65 |  | 14 (17) | 24 (13) | 8 (29) | 0.66 |  | -27 (23) | 0.11 |
| **Firmicutes** |  | 1590 (208) | 1604 (198) | 75 (214) | 0.34 |  | 1613 (290) | 1697 (213) | 10 (251) | 0.84 |  | 78 (264) | 0.18 |
| *Anaerostipes* | | 40 (27) | 53 (39) | 10 (37) | 0.80 |  | 60 (41) | 89 (56) | 27 (62) | 0.27 |  | -11 (71) | 0.45 |
| *Blautia* |  | 80 (47) | 80 (34) | -6 (37) | 0.59 |  | 93 (44) | 79 (40) | -22 (36) | 0.31 |  | 13 (54) | 0.23 |
| *Phascolarctobacterium* | | 85 (89) | 53 (63) | -34 (83) | 0.34 |  | 69 (72) | 64 (90) | -11 (73) | 0.62 |  | -22 (93) | 0.24 |
| *Dorea* |  | 53 (58) | 39 (17) | -21 (63) | 0.78 |  | 54 (57) | 40 (35) | -3 (32) | 0.77 |  | -17 (50) | 0.87 |
| *Faecalibacterium* | | 164 (114) | 210 (117) | 56 (120) | 0.32 |  | 170 (112) | 189 (126) | 10 (99) | 0.84 |  | 52 (146) | 0.17 |
| *Subdoligranulum* | | 105 (61) | 115 (73) | 5 (67) | 0.65 |  | 112 (70) | 119 (91) | 4 (59) | 0.82 |  | 1 (71) | 0.79 |
| **Proteobacteria** | | 85 (69) | 105 (71) | 21 (11) | 0.48 |  | 91 (81) | 90 (69) | 25 (56) | 0.32 |  | -5 (61) | 0.07 |
| **Tenericutes** |  | 30 (57) | 40 (45) | 2 (4) | 0.41 |  | 22 (28) | 35 (45) | 20 (24) | 0.72 |  | -16 (17) | 0.71 |
| **Verrucomicrobia** | | 113 (103) | 133 (120) | 37 (87) | 0.95 |  | 108 (104) | 91 (94) | 15 (74) | 0.62 |  | 15 (94) | 0.51 |
| *Akkermansia* | | 99 (104) | 82 (107) | -5 (98) | 0.95 |  | 71 (92) | 84 (94) | 14 (74) | 0.62 |  | -20 (101) | 0.51 |
|  | |  |  |  |  |  |  |  |  |  |  |  |  |
| **α-Diversity** | |  |  |  |  |  |  |  |  |  |  |  |  |
| Number of OTUs | | 131 (21) | 133 (27) | 2.2 (18.1) | 0.42 |  | 134 (24) | 132 (21) | -2.8 (15.3) | 0.73 |  | 6.5 (27) | 0.76 |
| Shannon Index | | 3.7 (0.2) | 3.7 (0.3) | 0.0 (0.2) | 0.26 |  | 3.7 (0.2) | 3.8 (0.2) | 0.1 (0.2) | 0.52 |  | -0.1 (0.3) | 0.08 |

OTU, operational taxonomic unit. * denotes significant within period differences for inulin and placebo, # denotes a significant difference between pre to post change for inulin and placebo periods.
